# Pediatric Nutrition in Different Countries

**DOI:** 10.3390/nu15092138

**Published:** 2023-04-29

**Authors:** Alessia Salatto, Maria Immacolata Spagnuolo

**Affiliations:** Department of Pediatrics, University Federico II of Naples, Via Sergio Pansini 5, 80131 Naples, Italy; mispagnu@unina.it

In this Special Issue, titled “Pediatric Nutrition in Different Countries”, we give concise and straightforward information on the nutritional habits of children in different countries worldwide.

It has long been known that the period from conception to the age of two years represents an important window of opportunity for ensuring the long-term health status of children. The intake of a wide variety of nutritious foods and proper energy intake in the early years of life are essential to reduce the risk of diet-related noncommunicable diseases. Until the age of 6 months, breast milk provides all the nutritional requirements for the infant. Controversies about proper nutrition begin in the weaning period. One of the articles in this Special Issue provides an index of complementary feeding utility based on recommendations from a longitudinal study of parents and children (ALSPAC). The study involved the completion of a questionnaire on the eating habits of a large cohort of Spanish children at various ages (2–3, 6, 12, 24–36 months), modeled on the PANCAKE pilot study to assess nutrients, food intake and consumption among children in Europe. This study showed that children evaluated at 12, 24 and 36 months had a low intake of fruits and vegetables and an excess of meat. Furthermore, the results allowed for the hypothesis that lower energy intake and the introduction at 6 months of certain food groups, such as vegetables, are associated with a better MDS (Mediterranean diet score) at 24 and 36 months, respectively. Additionally, a relevant finding was vitamin D deficiency in infants, the reversal of which could be considered a nutritional target in children over 12 months of age. This Spanish study therefore shows the importance of introducing certain foods, such as vegetables and fruits, from the beginning of the weaning period in preference to convenience foods in order to increase adherence to the Mediterranean diet in children [1].

Although we aim, over time, to fine-tune a child’s nutrition during the first three years of life, so that it can have a positive impact on his or her future health, what remains a certainty is the importance of exclusive breastfeeding during the first 6 months of life. Indeed, breastfed babies are less likely to become obese or overweight when they grow up, have a lower risk of noncommunicable diseases such as diabetes, show lower rates of infection (e.g., middle ear infection), and perform better on intelligence tests. Mothers who breastfeed also have benefits, such as reduced risks of ovarian cancer and type 2 diabetes. However, Alexandra Wolf-Spitzer’s Austrian group shows us that the WHO European Region has the lowest exclusive breastfeeding rates in the world at the age of 6 months. The Austrian SUKIE-Study (SUKIE: Säuglings und Kinderernährung), involving 1214 mothers, underlined how supporting mothers during pregnancy and in the first year of the child’s life is a key element for the total duration of breastfeeding and exclusive breastfeeding. This is equally as important as facilities such as baby-friendly hospitals, post-breastfeeding care and long-term maternal support facilities. However, these are not the only factors that affect the success of breastfeeding. Obese, smoking and low-income women would, in fact, be less willing to prolong breastfeeding and exclusive breastfeeding times [2].

It is therefore clear that the food and nutritional habits of children, especially children under 6 months, are strongly conditioned by the socio-cultural and economic context in which they grow up. Indeed, although the rate of breastfeeding and exclusive breastfeeding in Austria and the rest of Europe is low compared to the rest of the world (in Austria, the current breastfeeding initiation rate is 97.5%, but drops to 40.8% after 1 year of the child’s life), and Austrian guidelines recommend exclusive breastfeeding for about six months, among Syrian refugee babies in Greater Beirut, Lebanon, under six months it is even lower. For these and other reasons related to the precarious living conditions of mothers and children in this war zone, of the 114 children included in the study presented in this Special Issue, one fifth of them had anemia, 20.5%, and 9.6% were malnourished.

A significantly higher proportion of non-exclusively breastfed infants had fevers and infections and were on medications. These data confirm the importance of adequate nutrition in the first months of life, and of breastfeeding and exclusive breastfeeding [3].

Iron deficiency anemia appears to be increasingly central to the nutrient deficiencies of developing children. In Indonesia, eleven experts were invited to participate in a virtual meeting to discuss the current situation and the interventions available to prevent the problem. The Southeast Asia Nutrition Survey (SEANUTS) result found an even higher prevalence of anemia: nearly 55% among children under two years of age, and about 15% among children between two and twelve years old. In addition to affecting children, mainly in these age groups, anemia affects young women who would thus be exposed to reduced work productivity, an increased risk of infections, preterm birth, poor neonatal outcome, and even maternal mortality. The links between maternal anemia and offspring, where there is a positive correlation of the maternal hemoglobin level with the iron status of the child, are discussed in this Special Issue.

The etiology of anemia is diverse and complex. Various factors contribute to iron deficiency anemia (IDA), the most frequent form of anemia, such as a poor intake of iron-rich foods, an increased need associated with pregnancy, breastfeeding, or a rapid growth period in adolescence, and low absorption rates in chronic diseases. The insufficient availability of meat, fish or poultry is probably one of the main causes of iron deficiency in developing countries. Among the articles in this Special Issue, one tells how an iron supplementation program aimed at adolescents and pregnant women has been widely implemented in Indonesia [4].

The Japanese government also initiated a two-stage national health promotion scheme called Health Japan 21 in 2000 and 2012, which further promoted nutrition education for children at the national level, involving local governments and schools. The goal of this policy was to ensure the health of future generations by increasing the number of children who eat three adequate meals a day, or by increasing the frequency of family meals. This study shows the importance of school nutritional education and family habits in influencing young children’s nutrition choices [5].
